# Artemisinin Analogue SM934 Ameliorates Murine Experimental Autoimmune Encephalomyelitis through Enhancing the Expansion and Functions of Regulatory T Cell

**DOI:** 10.1371/journal.pone.0074108

**Published:** 2013-08-29

**Authors:** Xin Li, Tian-Tian Li, Xiao-Hui Zhang, Li-Fei Hou, Xiao-Qian Yang, Feng-Hua Zhu, Wei Tang, Jian-Ping Zuo

**Affiliations:** 1 Laboratory of Immunopharmacology, State Key Laboratory of Drug Research Shanghai Institute of Materia Medica, Chinese Academy of Sciences, Shanghai, People’s Republic of China; 2 Laboratory of Immunology and Virology, Shanghai University of Traditional Chinese Medicine, Shanghai, People's Republic of China; Wayne State University, United States of America

## Abstract

**Background:**

Artemisinin analogue SM934 was previously reported to possess immunosuppressive properties. The aim of this study was to determine the effects and the underlying mechanisms of SM934 in murine experimental autoimmune encephalomyelitis (EAE).

**Methods:**

Female C57BL/6 mice immunized with MOG_35–55_ were treated with or without SM934, then the clinical scores and other relevant parameters were assessed. Th1, Th17 and regulatory T (Treg) cell profiles were determined through ELISA, qRT-PCR, flow cytometry and BrdU incorporation assay. The effects of SM934 on Th1, Th17 and Treg cells differentiation were explored through intracellular staining and flow cytometry examination.

**Results:**

*In vivo*, administration of SM934 significantly inhibited the development of EAE and suppressed the elevation of serum IL-17. *Ex vivo*, upon antigen-recall stimulation, IL-2, IFN-γ, IL-17 and IL-6 production were decreased, whereas IL-10 and TGF-β production were increased from the splenocytes isolated from SM934-treated mice. Consistently, both flow cytometry and qRT-PCR results showed that SM934 treatment significantly increased the Treg, while strongly suppressed the Th17 and Th1, responses in the peripheral. Furthermore, in the spinal lesion, SM934 treatment dramatically decreased the infiltration of CD4^+^ T cells, within which the Treg cells percentage was enlarged, whereas the Th17, but not Th1 percentage, was significantly decreased comparing with the vehicle-treated groups. Finally, both BrdU incorporation and *in vitro* Treg differentiation assays revealed that SM934 treatment could directly promote the expansion of Treg cells *in vivo* and *in vitro*.

**Conclusion:**

Taken together, this study demonstrated that SM934 treatment could ameliorate the murine EAE disease, which might be mediated by inducing Treg differentiation and expansion.

## Introduction

Multiple sclerosis (MS) is an inflammatory disease in which the myelin sheath around the axons of brain and spinal cord are damaged that lead to demyelination. EAE, a classical animal model of MS, which is characterized by abnormal inflammatory cells infiltrating into the central nervous system (CNS), thus initiating the lesion formation and finally the demyelination of neuron axon [1]. Numerous types of cells play varied and complex roles during the development of MS/EAE. Initially, it was recognized that IFN-γ-producing Th1 cells were predominant pathogenic cells in MS and EAE [2], and its key role was also supported by establishing EAE model upon adoptive transfer of IL-12 polarized Th1 cells [3]. However, mice deficient in Th1-associated molecules, such as IFN-γ[4], IL-12Rβ2 [5] or IL-12p35 [6] were more susceptible to EAE, while IL-12p40 deficient mice were resistant to disease [7], which challenged the predominance of Th1 in EAE pathogenesis. The discovery of IL-23, which shares the IL-12p40 common subunit with IL-12, together with the identification of IL-17-producing CD4^+^ T cells (Th17), deepened the understanding of pathogenesis in EAE. Mice deficient in IL-23p19 subunit are completely resistant to EAE [8] and display defects in the Th17 compartment [9]. In addition, IL-23 treated myelin-specific CD4^+^ T cells are more encephalitogenic [10]. IL-17 deficient mice also showed partially insensitive to EAE [11]. Th17 differentiation during EAE also depends on IL-6. In the absence of IL-6, TGF-β could induce naïve CD4^+^ T cell differentiate into regulatory T cell, but in the presence of IL-6, naïve CD4^+^ T cell could differentiate into Th17, and IL-6 deficient mice are also completely resistant to EAE [12], [13]. These reports demonstrated that Th17 cells, as well as Th1 cells, play a pivotal pathogenic role in the development of EAE.

Treg cells is another important subpopulation of CD4^+^ T cells that has a critical role in maintaining immune homeostasis and tolerance. Forkhead box P3 (Foxp3) is essential for Treg cells development and function. Mice or humans carrying genetic mutations in Foxp3, usually associated with defective Treg cells functions, suffered from severe lympho-proliferative disorders and immune diseases [14]–[17]. Further investigations showed that Treg cells exerted their major role in the maintenance of immune-tolerance through several mechanisms including secretion of inhibitory molecules, suppression of antigen presenting cells function, induction of cytolysis and effector cells metabolic disruption [18]. The balance between effector T cells (Teff) and regulatory T cells with a common specificity can therefore profoundly influence the outcome of antigen encounter [19]. Previous studies in the EAE model demonstrated that adoptive transfer of Treg cells conferred significant protection from clinical EAE which was associated with normal activation of Th1 and increased production of Th2 cytokines, and decreased infiltration into the CNS [20]. Drugs enhancing Treg differentiation or inhibiting Th17 development, for example, Rapamycin, could ameliorate EAE [21].

Artemisinin (Qinghaosu), a widely used anti-malaria drug, was reported possessing immunosuppressive effects *in vitro* and *in vivo* [22]. However, most of the artemisinin analogues in the clinical utilise have poor water solubility, which may limit their clinical application for oral administration. SM934 is a novel water-soluble artemisinin derivative indentified by our laboratories from a series of new compounds derived from artemisinin, and showed promising immunosuppressive activities both *in vitro* and *in vivo* [23]. Our previous reports have demonstrated that SM934 could modulate autoimmune responses in systemic lupus erythematosus (SLE) via enhancing the secretion of IL-10 by macrophages to repress both Th1 and Th17 responses [24], [25]. SM934 treatment could also relatively increased the percentage of Treg cells in lupus-prone mice. In the current study, we investigated the effects of SM934 on murine EAE model, and explored the underlying immunological mechanisms. The results showed that SM934 treatment significantly ameliorated the EAE disease by suppressing Th1 and Th17 responses, enhancing Treg cells differentiation and expansion *in vivo*. The current study demonstrated that artemisinin analogues are promising immuno-regulatory agents for autoimmune disease therapy by modulating Treg/Teff balance, especially promoting the expansion and functions of Treg cells.

## Results

### SM934 treatment inhibited the development of EAE in MOG-immunized C57BL/6 mice

**Figure 1:**
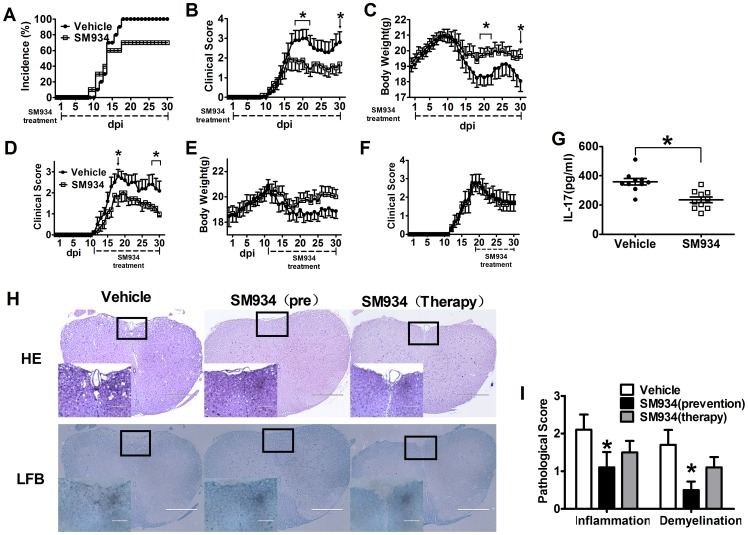
SM934 treatment inhibited the development of EAE in MOG-immunized C57BL/6 mice. Active EAE was induced in female C57BL/6 mice by immunization with MOG_35−55_. The mice were orally treated with vehicle or SM934 (10 mg/kg/day) from day 1 post-immunization as detailed under Materials and methods. Mice were monitored for signs of EAE, and the results for all mice, both healthy and sick, were presented as (A) incidence of disease, (B) mean clinical score ± SEM, and (C) body weight. Immunized mice were treated with vehicle or SM934 (10 mg/kg/day) from day 11 post-immunization, and the clinical score (D) and body weight (E) were monitored. Immunized mice were treated with vehicle or SM934 (10 mg/kg/day) from day 18 post-immunization, and the clinical score (F) were monitored. (G) Serum from both groups of mice were collected at the peak of disease (day 18 p.i.), and measured IL-17 level by ELISA, each dot represents one mouse. n  =  10 in each group. (H) Spinal cords were collected from EAE mice treated with vehicle or SM934, treated from day 1 p.i. (pre) or treated from day 11 p.i. (therapy). The sections were stained with H&E and Luxol fast blue to assess inflammation and demyelination. (I) Pathological scores including inflammation and demyelination were analyzed and shown with bar graph. Results were expressed as mean ± SEM. *, p<0.05, compared with vehicle control. Three independent experiments were performed with similar results.

To investigate the protective effects of SM934 against the development of EAE, we immunized female C57BL/6 mice with MOG_35−55_ peptide emulsified with CFA, followed by oral administration of SM934 (10 mg/kg) or vehicle 1 day after immunization. All mice (100%) in the vehicle treated group developed severe EAE at approximately day 18 to 20. In contrast, less than 70% of mice treated with SM934 showed mild signs of disease (Figure 1A and Table 1). Besides, mice treated with SM934 exhibited significant reduction in the severity of EAE. On day 21, when EAE clinical score reached the peak, the mean score of SM934-treated group was 1.6 ± 1.2, in comparison with 3.0 ± 1.4 for the vehicle group (Figure 1B and Table 1). SM934 treatment also markedly prevented the loss of body weight in EAE mice (Figure 1C). To determine the therapeutic effect of SM934, the EAE mice were also treated from the day of disease onset (day 11 p.i.), the treatment of SM934 from the disease onset could also reduce EAE severity (Figure 1D and Table 1) and the loss of body weight (Figure 1E). However, the treatment of SM934 from the disease peak did not significantly reduce the EAE severity (Figure 1E and Table 1).

**Table 1:** Clinical features of EAE in the administration of Vehicle or SM934.

	Preventive treatment (n = 30)				Therapeutical treatment from onset (n = 30)		Therapeutical treatment from peak (n = 30)	
Group	Incidence %)	Average day of onset	Average day of peak	Mean maximal score	Average day of peak	Mean maximal score	Average day of peak	Mean maximal score
Vehicle	100%	13.5±0.33	17.8±0.42	3.37±0.20	18.3±0.61	3.57±0.14	19.0±0.77	3.3±0.17
SM934	63%	20.3±1.48***	22.4±1.20**	2.2±0.34*	20.9±0.74**	2.77±0.18**	18.4±0.47	3.1±0.23

Data were expressed as mean ± SEM, from three independent experiments,

* *P* <0.05, ** *P* < 0.01, ***p<0.001 versus vehicle.

Considering the pivotal role of IL-17 in EAE development, CNS inflammation and chemotaxis in encephalomyelitis [26], we further analyzed serum IL-17 levels in EAE mice. As shown in Figure 1G, serum IL-17 levels were significantly decreased by SM934 treatment.

Histological analysis of spinal cord tissue sections from vehicle and SM934 treated mice were carried out. Typical inflammation (HE stain) and demyelination (Luxol fast blue) were observed in vehicle treated mice, and SM934 treatment remarkably attenuated CNS inflammation and demyelination (Figure 1H and I). These results suggested that SM934 reduced the duration and clinical severity, and inhibited inflammatory responses in EAE mice.

### SM934 treatment suppressed MOG-specific pro-inflammatory cytokines and enhanced anti-inflammatory cytokines production in splenocytes from EAE mice

**Figure 2:**
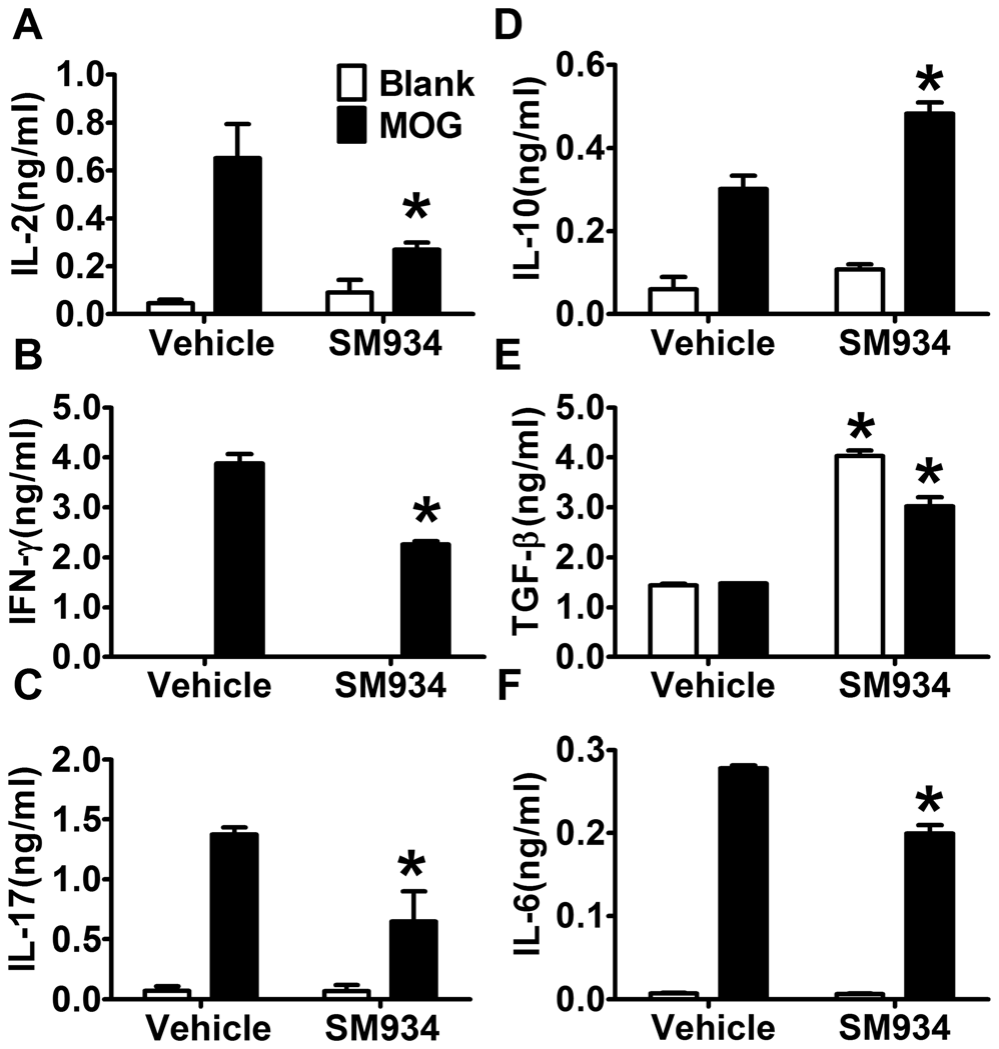
SM934 treatment suppressed MOG-specific pro-inflammatory cytokines and enhanced MOG-specific anti-inflammatory cytokines production in splenocytes from EAE mice. Splenocytes from immunized mice treated with vehicle or SM934 (10 mice per group) were isolated at the peak of disease (day 18 p.i.), re-stimulated with MOG_35–55_ (10 µg/ml) for 72 hours. Culture supernatants were collected and indicated cytokine levels were measured by ELISA. Results were expressed as mean ± SEM. *, p<0.05 compared with vehicle control. Three independent experiments were performed with similar results.

To investigate the immunological profiles after SM934 treatment in EAE, splenocytes from sick mice at the peak of disease (day 18 p.i.) were separated and re-stimulated with MOG_35–55_ for 72 hours, and the cytokine production profiles were analyzed. As shown in Figure 2, Th1 and Th17 associated cytokines, IL-2, IFN-γ and IL-17 production were significantly decreased by SM934 treatment. IL-10, the most important immunosuppressive cytokine, was significantly up-regulated in SM934 treated mice. Although TGF-β is commonly considered as an immune suppressive cytokine, its function is still duplex. It is generally accepted that TGF-β alone induces Treg cells, and maintains Treg cells survival. But in contrast, TGF-β together with IL-6, could induce Th17 differentiation [13]. The results showed higher level of TGF-β but lower level of IL-6 in SM934 treated group, which suggested that SM934 treatment favors Treg differentiation, but constrains Th17 development.

### Effects of SM934 on the polarization of CD4^+^ T cells in EAE mice

**Figure 3:**
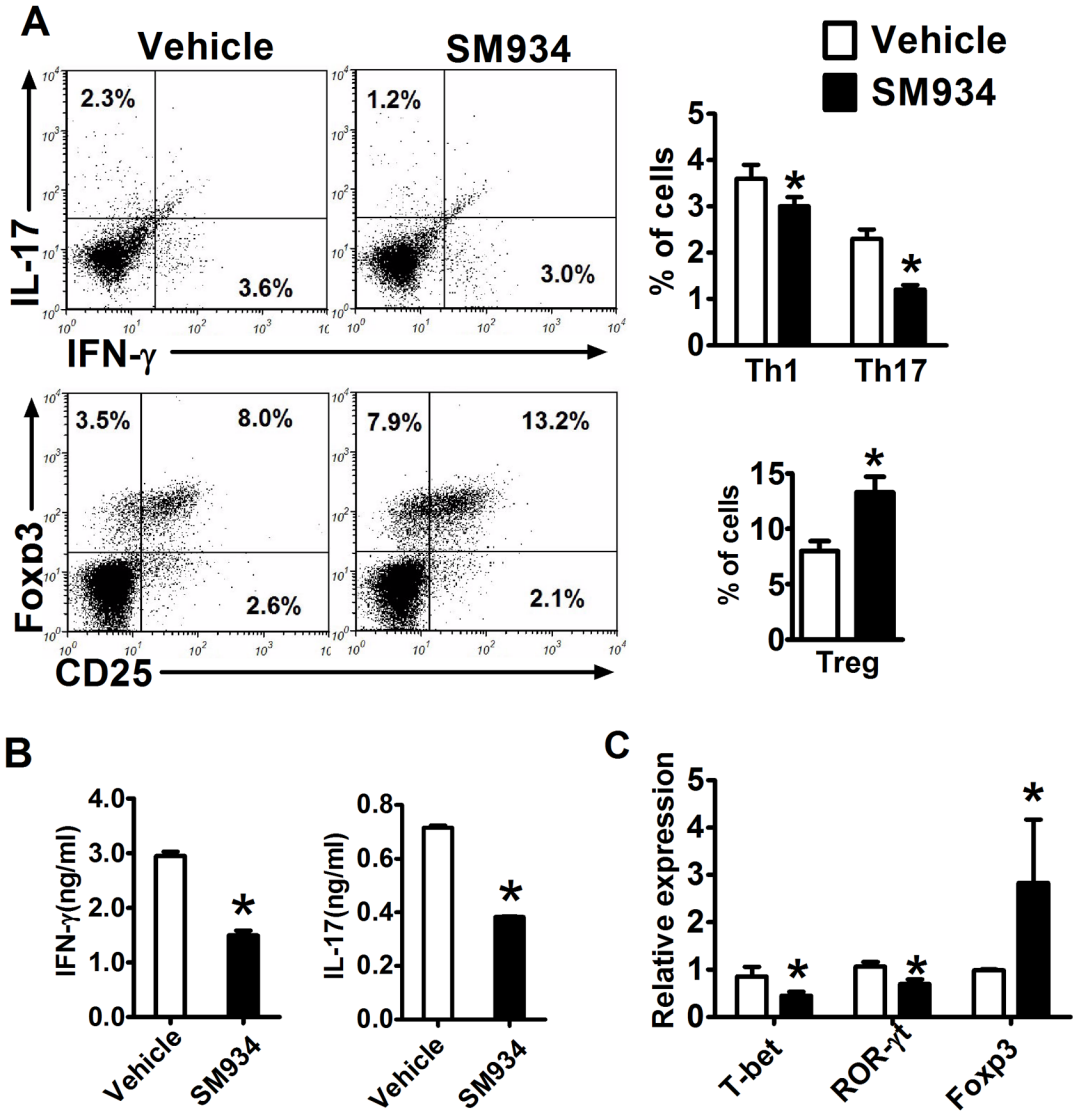
Effects of SM934 on the polarization of CD4^+^ T cells in EAE mice. Splenocytes from immunized mice treated with vehicle or SM934 were isolated at the peak of disease (day 18 p.i.) and analyzed. (A) The percentage of Th1, Th17 and Treg cells in the CD4^+^ gate were analyzed by flow cytometry, values in the bar graphs are the mean ± SEM (n = 4). (B) Splenocytes were re-stimulated with IL-12 (20 ng/ml) or IL-23 (20 ng/ml) for 72 hours, and supernatants were collected to measure IFN-γ and IL-17 levels, respectively. (C) RNA from splenocytes was analyzed by real-time PCR for expression of T-bet, RORγt and Foxp3. Results were expressed as mean ± SEM. *, p<0.05 compared with vehicle control. Three independent experiments were performed with similar results.

To further investigate the possible role of SM934 in helper T cell polarization, we analyzed the CD4^+^ T cell subsets in spleens from EAE mice at the peak of disease. In accordance with *ex vivo* results, there was a significant decrease of the percentage of IFN-γ^+^ producing CD4^+^ T cell (Th1) and IL-17^+^ producing CD4^+^ T cell (Th17), but an increase of the percentage of CD4^+^CD25^+^Foxp3^+^ T cells (Treg) in splenocytes (Figure 3A).

We further re-stimulated splenocytes with IL-12 or IL-23 to analyze IFN-γ or IL-17 production, respectively. As shown in Figure 3B, lower levels of IFN-γ production upon IL-12, and IL-17 production upon IL-23 stimulation were observed in splenocytes from SM934 treated mice. In addition, the expression of Th1-, Th17- and Treg- specific transcription factors in splenocytes were examined by qRT-PCR. The results presented in Figure 3C indicated that SM934 treatment slightly reduced T-bet (Th1) and ROR-γt (Th17) expression, but enhanced Foxp3 expression.

### SM934 reduced encephalitogenic cells infiltrating into the CNS

**Figure 4:**
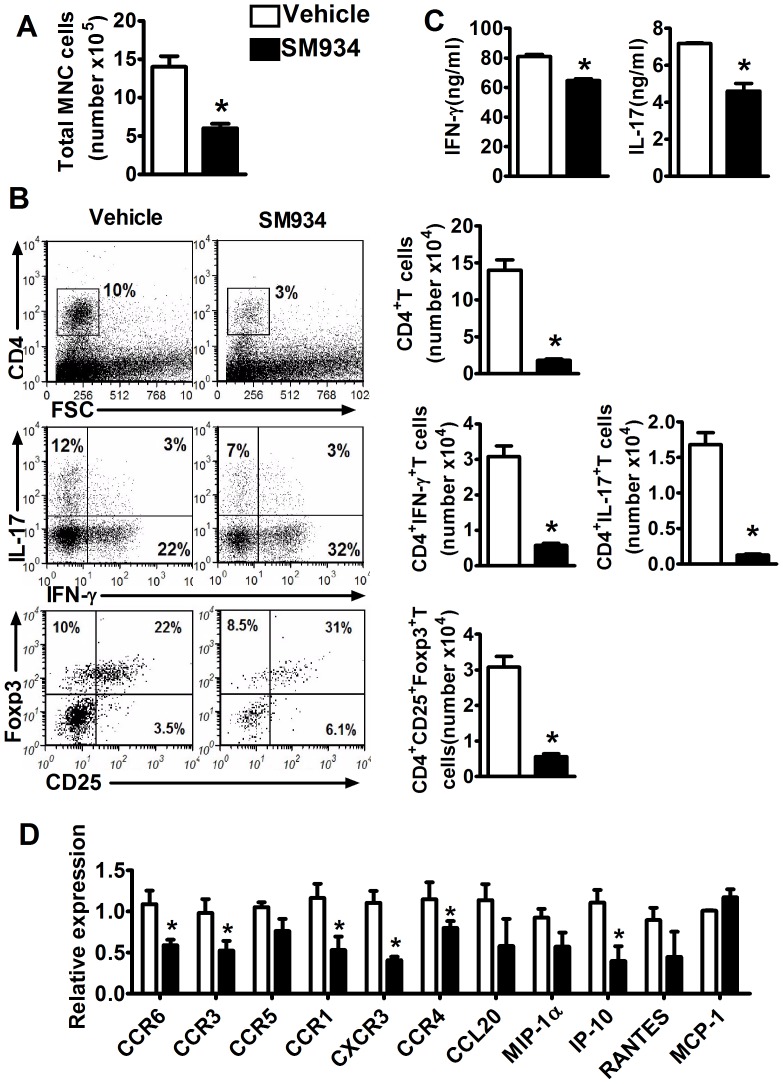
SM934 reduced encephalitogenic cells infiltrating into the CNS. Spinal cord were detached from EAE mice at the peak of disease (day 18), infiltrated mononuclear cells were isolated by density gradient centrifugation, (A) cell number were counted, (B) intracellular IFN-γ, IL-17 and Foxp3 in the CD4^+^ T cells were analyzed by flow cytometry, absolute number of CD4^+^IFN-γ^+^, CD4^+^IL-17^+^, CD4^+^CD25^+^Foxp3^+^ cells were calculated. (C) Isolated MNC were stimulated with IL-12 (20 ng/ml) or IL-23 (20 ng/ml) for 72 hours, supernatants were collected to measure IFN-γ and IL-17 levels, respectively. (D) Spinal cord were isolated and analyzed by real-time PCR for expression of indicated chemokines and receptors. Results were expressed as mean ± SEM. *, p<0.05 compared with vehicle control. Three independent experiments were performed with similar results.

To confirm the activity of SM934 on T cell subpopulations in regional CNS, the spinal cord was detached and analyzed for the cell infiltration at the peak of disease. The infiltrated mononuclear cells (MNC) were isolated, the absolute number was counted and the cell population was analyzed by flow cytometry. The results showed that MNC number in the spinal cord from SM934 treated mice was dramatically decreased, with corresponding reductions in the percentage and absolute count of CD4^+^ T cells (Figure 4A&B). Furthermore, in the CD4^+^ T cells, a similar decline in the percentage and absolute number of IL-17^+^ cells (Th17) was observed. As for IFN-γ^+^CD4^+^ T cells (Th1), the percentage was higher in SM934 treated group, but still had the sharply reduction of absolute count. Oppositely, the absolute number of Treg cells was lower, even with a higher percentage in SM934 treatment mice, which was also due to the declined contents of MNC and CD4^+^ T cells (Figure 4B). The isolated MNC were further incubated with IL-12 or IL-23 and tested the cytokines secretion. The results showed that IFN-γ and IL-17 production in MNC from SM934 treated mice were significantly decreased in response to IL-12 or IL-23 (Figure 4C).

Chemokines and their receptors play crucial roles in the trafficking of leukocytes and are of particular interest in the context of the unique inflammatory responses elicited in the CNS. In EAE, there is a tight correlation between expression of chemokines and the distribution of leukocytes infiltrating into the CNS and the development of disease [27]. Thus we analyzed the expression of chemokines and their receptors in the spinal cord lesion. As shown in Figure 4D, the expression CCL20 and its receptor CCR6, which mediating Th17 and Treg cells infiltrating into the CNS, were decreased by SM934 treatment, suggesting decreased Th17 and Treg cells infiltrating into CNS. The chemokines RANTES, MIP-1α, IP-10 and their receptors CCR1, CCR3, CCR5, CXCR3, which can mediate different types of inflammatory cells including T cells, B cells, macrophages, dendritic cells, granulocytes infiltrating into the CNS [28], were also decreased to varying degrees after the treatment of SM934. However, the expression of MCP-1, which also could mediate T cells, B cells, macrophages infiltrating, was not seen any influence by SM934 treatment, with a lower level in its receptor CCR4 expression.

### SM934 specifically induced regulatory T cells expansion

**Figure 5:**
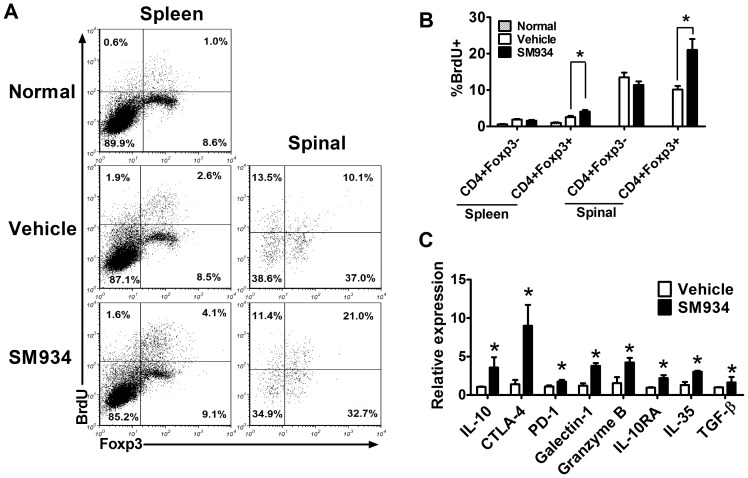
SM934 treatment enhanced Treg cells expansion in EAE mice. (A) Vehicle and SM934 treated mice were given daily i.p. injection of BrdU for 3 days from day 17 p.i., splenocytes and MNC in spinal cord were isolated and analyzed for BrdU incorporation. The proliferative profile was evaluated in CD4^+^Foxp3^−^, CD4^+^Foxp3^+^ splenocytes, and CD4^+^Foxp3^−^, CD4^+^Foxp3^+^ from spinal cord. (B) Percentage of CD4^+^Foxp3^−^, CD4^+^Foxp3^+^ splenocytes, and CD4^+^Foxp3^−^, CD4^+^Foxp3^+^ from spinal cord are expressed as mean ± SEM (n = 4). (C) CD4^+^ T cells were purified by immunomagnetic negative selection from splenocytes, indicated genes expression was analyzed by real-time PCR. Results were expressed as mean ± SEM. *, p<0.05 compared with vehicle control. Three independent experiments were performed with similar results.

To further determine whether SM934 directly influence the Treg cells *in vivo*, BrdU incorporation assay was performed in EAE mice and the proliferation of Teff and Treg cells was compared. Results presented in Figure 5A showed that SM934 significantly enhanced Treg cells (CD4^+^Foxp3^+^) proliferation both in the peripheral and CNS, while showing no effect on the proliferation of Teff (CD4^+^Foxp3^−^).

As we known, Treg cells exerted suppressive functions through multiple ways. First of all, Treg cells secreted suppressive cytokines. IL-10 plays an important role in Treg cells mediated suppression of inflammation, blocking IL-10 or IL-10 deficient abrogated the protective effect of Treg cells [29], [30]. In addition, IL-10RA is also critical for the suppressive effect on Th17 of Treg cells [31]. Treg cells can also produce high amounts of TGF-β, and blocking TGF-β partially abrogated its suppressive effects of T cell proliferation *in vitro*, those suggests that Treg-produced TGF-β controls autoimmunity[32]. IL-35 is a recently discovered cytokine implicated in Treg-mediated suppression and was shown to directly inhibit Teff proliferation [33]. Secondly, suppressive ligands expressed on the surface of Treg cells also play the suppressive functions. CTLA-4 is constitutively expressed on Treg cells, could bind to CD80 and CD86 competing with CD28, CTLA-4 deficient Treg cells are less suppressive than wild type Treg cells [34]. PD-1 is another surface suppressive ligand binding to PD-L1 and PD-L2, although it is not specifically expressed on Treg cells, it is important for Treg cells suppressive abilities [35]. Galectin-1 is preferentially expressed in Treg cells, and binds to many glycoproteins including CD45, CD43 and CD7, and it could induce cell cycle arrest and apoptosis and inhibit the production of proinflammatory cytokines[18]. Finally, activation of Treg cells also results in upregulation of granzyme B expression and induce responder cells apoptosis [36]. To determine whether SM934 treatment influenced the immune suppressive functions of Treg cells, we analyzed the expression of the above mentioned Treg function-associated factors in purified CD4^+^ T cells from splenocytes of EAE mice. As shown in Figure 5B, the expression of indicated Treg suppressive factors was up-regulated in SM934 group, indicating that SM934 treatment enhanced the suppressive functions of Treg cells.

### SM934 treatment enhanced Treg differentiation *in vitro*

**Figure 6:**
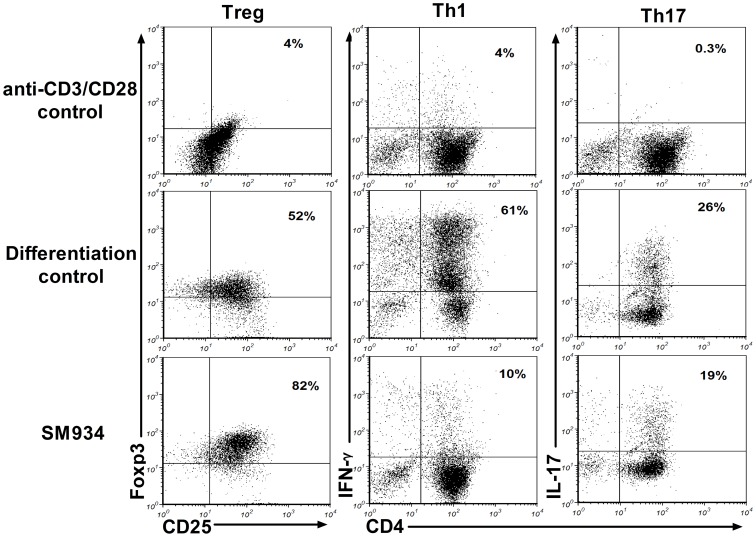
SM934 regulated Th cell differentiation *in vitro*. Naïve CD4^+^ T cells in spleens were purified by immunomagnetic positive selection from normal C57BL/6 mice, and cultured in Treg, Th1, Th17 cells differentiation conditions for 96 hours, with or without SM934 (1 µM). Frequencies of Treg, Th1 and Th17 were quantified using flow cytometry. Three independent experiments were performed with similar results.

Our previous reports indicated that SM934 inhibited Th1 and Th17 differentiation *in vitro*, but did not influence Treg differentiation at the concentration of 10 µM, however, the dose was much higher than its IC_50_ value at approximately 2 µM [23]. To clear away the possible interference of suppressing T cell proliferation, we evaluated the effect of SM934 on naive CD4^+^ T cells differentiation at the dose of 1 µM, lower than its IC_50_ value. In the *in vitro* differentiation system, SM934 significantly promoted Treg differentiation at lower concentration, but the inhibitory effects on Th1 and Th17 differentiation were still remaining (Figure 6).

## Discussion

Artemisinin and its derivatives, such as Artesunate and Dihydroartemisinin, have been reported to have immune-regulatory effects for many years [37]–[39]. SM934, a water soluble artemisinin analogue, exerted immune-regulatory effects both *in vitro* and *in vivo*. In our study, we observed the effect of SM934 on EAE model and explored its mechanism of action. Our results demonstrated that the severity and the development of EAE could be inhibited by both SM934 treatment from the day of immunization and the onset of the disease. However, the therapeutic effect from the peak of disease was not satisfactory. Similarly, both Zhao et al and Wang et al showed that dihydroartemisinin and SM933, another artemisinin analogue, could ameliorate murine EAE model respectively, when the administration at the beginning and the onset of the disease (no data about treating at the peak of disease showed in both papers)[38], [39]. Before the onset of EAE, the helper T cells were firstly activated by the activation of myelin antigen. By the help of T cells, macrophages and neutrophils were accumulated in the lesion and induce the inflammatory responses. At this stage, the role of helper T cells was less crucial. [40]. In this stage, the immunoregulatory agents targeting the balance of Teff and Treg cells, such as artemisinin analogues, might be less effective. Considering the difference between human MS and murine EAE model, especially the alleviation/relapse happens in human patients, while the T cells could also crucial in the relapse phase, we still deem SM934 also meaningful for clinical MS patient. Besides that, effects of SM934 on remit-relapse EAE induced in SJL mice will be performed to confirm the therapeutical effects for the usage in MS treatment in our further study.

Mechanism study found that SM934 treatment significantly reduced the inflammation and demyelination in active EAE model, simultaneously reduced Th1 and Th17 accumulation both in the peripheral immune system and the CNS lesion, up-regulated Treg cells percentage. Further study demonstrated that SM934 treatment directly enhanced Treg cells expansion and differentiation. Though the general effects and mechanisms of these artemisinin analogues are similar, subtle differences turned up between SM934 and dihydroartemisinin because of their difference of molecular structures and water solubility. *In vivo*, SM934 reduced the relative percentage of Th17 in CNS infiltrated MNC, but increased the Th1 relative percentage; in contrast, dihydroartemisinin both markedly reduced Th1 and Th17 percentage. *In vitro*, SM934 almost completely inhibited Th1 differentiation, and partially inhibited Th17 differentiation. Dihydroartemisinin exerted minor inhibition of Th1, but inhibited Th17 almost completely. But in common, SM934 and dihydroartemisinin both effectly enhanced Treg differentiation. Actually, our previous studies have shown that differenct artemisinin analogues SM934 and artemether possessed different immunoregulatory properties[23], [41]. Most of all, what we could conclude here is that inducing the expansion of Treg cells is the common feature shared by artemisinin analogues to date. Further investigations should be made and cautions should be put on the different artemisinin analogues, regarding the possible clinical using as immunoregulatory agents.

It is well known that Treg cells play an essential role in regulating autoimmune responses. In EAE model, Treg cells were reported for many approaches to prevent inflammation. Several studies had shown that Treg cells depletion prior to EAE induction increased the severity of disease, and IFN-γ, IL-6 and IL-17 production were enhanced, which could indicate that Treg cells suppress the expansion of autoreactive effectors [42]. Suppressive functions and number of Treg cells were impaired during autoimmune diseases. In multiple sclerosis, the quantity and function of Treg cells were both decreased [43]. In the course of murine EAE immunization, pertussis toxin was used for selectively reducing the number and function of Treg cells *in vivo* to enhance EAE severity [44]. During the EAE progression, inflammatory cells, such as Th1, Th17 and macrophages were accumulated in the CNS lesion[45], [46]. On the other side, regulatory T cells were also accumulated in the lesions which were critical for regulating the inflammatory reactions. Neuron-T cell interaction results in the generation of Treg cells, and Treg cells could also be converted from activated encephalitogenic T cells during CNS inflammation[47]. In addition, Treg cells entering the CNS in response to inflammation could initiate a dramatic proliferative burst [48]. Upon purification and transfer, CNS-derived and neuron-induced Treg cells are capable of preventing progression of EAE. For the recovery phase of EAE, Treg cells were the major source of IL-10 in the CNS, and IL-10 production is crucial for full recovery[49]. Although Treg cells can be induced to produce IL-17 by *in vitro* exposure to pro-inflammatory cytokines, Treg cells in the inflamed CNS are insensitive to IL-6 driven IL-17 production [50]. Enhancing Treg cells or regulation the balance of Treg/Teff cells were used as therapeutic methods for autoimmune diseases. Rapamycin, which can selectively enhance Treg differentiation and suppress pathogenic Th17 differentiation, was used for curing autoimmune diseases and preventing organ transplantation rejection in clinic [51], [52]. Retinoic acid, which could enhance Treg cells differentiation, was also described for the usage in the treatment of the autoimmune diseases animal model in research [53]. In addition, therapeutically expansion of Treg cells might be advantageous in autoimmunity. Intravenous immunoglobulin were reported for its controlling EAE effect through expanding Treg cells[54]. Pretreating with IL-2-mAb complexes renders the mice resistant to induction of EAE through selectively enhancing Treg cells expansion *in vivo* [55].

In our current study, we demonstrated that SM934 exert therapeutic effect on EAE for suppressing the progression and reversing the clinical and histological signs. Fewer cells arrived in the CNS after SM934 treatment. The disharmonious T cell subpopulation in EAE mice was restored to some extent, both in peripheral and CNS. The Treg cells population and functions were markedly promoted under the treatment. Further studies confirmed that SM934 treatment directly enhanced Treg cells expansion.

Collectively, our studies have demonstrated that SM934 treatment ameliorated the EAE severity by favoring Treg cells expansion and functions, indicating the immunosuppressive mechanisms of artemisinin analogue SM934.

## Materials and Methods

### Animal experiments

Female C57BL/6 mice were obtained from Shanghai Laboratory Animal Center of the Chinese Academy of Sciences, and were used at 8 to 10 weeks of age. All mice were housed under specific pathogen-free conditions. All experiments were performed according to the institutional ethical guidelines on animal care and approved by the Institute Animal Care and Use Committee at Shanghai Institute of Materia Medica.

### Induction, treatment, and clinical evaluation of EAE

The murine EAE model was produced as described previously [56]. Briefly, female C57BL/6 mice were immunized on day 0 by subcutaneous (*s.c.*) injection with 150 µl of an emulsion containning MOG_35–55_ CFA (Sigma-Aldrich) supplemented with Mycobacterium tuberculosis H37RA (Difco). The final dose of MOG_35–55_ and *Mycobacterium tuberculosis H37Ra* was 150 µg and 300 µg per mouse. These injections were distributed over the following sites: one along the midline of the back between the shoulders and two on either side of the midline on the lower back. Each mouse received an additional 400 ng of *Bordetella pertussis toxin* (List Biological Laboratory) by intraperitoneal (*i.p.*) injection in 200 µl of PBS on day 0 and day 2 post-immunization. Vehicle and SM934 were administered orally following the immunization and continued throughout the study (n = 10). The dose of SM934 (10 mg/kg) was chosen based on our earlier results of DTH and SLE experiments [23], [25]. Clinical assessment of EAE was performed daily and mice were scored for disease according to the following criteria: 0, no overt signs of disease; 1, limp tail or hind limb weakness but not both; 2, limp tail and hind limb weakness; 3, partial hind limb paralysis; 4, complete hind limb paralysis; 5, moribund state or dead.

### Histopathology

To assess the degree of CNS inflammation and demyelination, immunized C57BL/6 mice treated with vehicle or SM934 were anesthetized by pentobarbital sodium and perfused by intracardiac injection of PBS containing 4% paraformaldehyde. Paraffin-embedded 5 µm sections of spinal cord were stained with H&E and Luxol fast blue and then examined by light microscopy. Briefly, inflammation was scored as follows: 0, none; 1, a few inflammatory cells; 2, organization of perivascular infiltrates; 3, increasing severity of perivascular cuffing with extension into adjacent tissue. Demyelination was scored as follows: 0, none; 1, rare foci; 2, a few areas of demyelination; 3, large (confluent) areas of demyelination [57].

### MOG_35–55_-Specific immune responses

Ten mice in each group for considering the protective effect of SM934 in EAE model, and preventive treatment (treatment started at the day 1 p.i.) was performed with SM934 or vehicle. At the peak of disease (day 18 p.i.), all mice were divided into four subgroups (2, 2, 3, 3 mice were contained in each subgroup respectively), and sacrificed, cells of each subgroup were pooled together and *ex vivo* re-stimulation was performed. Splenocytes were cultured with or without 10 µg/ml MOG_35–55_ stimulation. Supernatants were harvested at the indicated times to measure cytokine levels by ELISA. Data were presented as the mean value and SEM of the four subgroups.

### Polyclonal and naïve CD4^+^ T cell purification

Polyclonal and naïve CD4^+^ T cells were isolated as described previously [25]. To acquire polyclonal CD4^+^ T cells, immunomagnetic negative selection was used by adding monoclonal antibody cocktails to deplete CD8^+^ cells, B220^+^ cells, CD11b^+^ cells, and I-A^+^ antigen presenting cells from splenocytes. The purity of isolated CD4+T cells was examined by flow cytometry and found to be consistently > 90%. To acquire naïve CD4^+^ T cells (CD4^+^CD44^−^CD62L^+^), anti-CD44 mAb (KM201) were added to the mAb cocktails of the immunomagnetic negative selection system to remove no-CD4^+^ cells and CD44^+^ cells. In both negative-selection cases, the remaining cells were then incubated with L3T4 Microbeads (Miltenyi Biotec) and positively selected. The purity of the resulting cells were determined by flow cytometry analysis and was consistently >95%.

### Isolation of spinal cord infiltrated mononuclear cell

Spinal cord infiltrated mononuclear cells were isolated as described previously [58]. The mice were anaesthetized with pentobarbital sodium and perfused with 20 ml of cold PBS. The spinal cords were extruded by flushing the vertebral canal with PBS and rinsed in PBS. Tissues were forced through 70 µM nylon cell strainers (BD Falcon), and then the spinal cord cell suspensions were incubated with collagenase (1 mg/ml) at 37°C for 30 min, and passed again through 70 µM nylon cell strainers to yield single-cell suspensions. CNS mononuclear cells were centrifuged (400×*g*) at room temperature for 20 min over discontinuous 30%/70% Percoll gradient (GE Healthcare). The MNC were counted and immediately analyzed by flow cytometry, or incubated for 72 h with IL-12 or IL-23 for later cytokine assay, or lysed by TRIzol Reagent (Invitrogen) for qRT-PCR analyze.

### Flow cytometry analysis

Surface marker and intracellular staining was conducted and analyzed according to our previous methods [25]. For intracellular cytokine staining, cells were incubated for 4 hours with phorbol 12-myristate 13-acetate (PMA, 50 ng/ml) and ionomycin (750 ng/ml) in the presence of Brefeldin A (BFA, 10 µg/ml, Sigma-Aldrich). At the end of incubation, suspended cells were collected and blocked with anti-mCD16/CD32 (2.4G2) mAb before extracellular staining for corresponding fluorescence-labeled surface antibodies. After surface staining, cells were fixed, permeabilized and stained for cytokines according to manufacturer’s instruction (Foxp3 Staining Buffer set was used).

The following reagents were used: FITC-, PE-, APC- and Pe-cy5-anti-mCD4 (GK1.5), FITC- and APC-anti-mIFN-γ (XMG1.2), PE-anti-mIL-17 (TC11-18H10), were all purchased from BD Pharmingen. Pe-Cy5- and PerCP-Cy5.5-anti-mFoxp3 (FJK-16s) were purchased from eBioscience. The Foxp3 Staining Buffer Set was purchased from eBioscience.

### BrdU incorporation assay in EAE mice

5-Bromo-2′-deoxyuridine (BrdU, Sigma-Aldrich) were injected i.p. daily at a dose of 2 mg per mouse continuously for 3 days from day 17 post immunization. Four hours after the last injection, mice were sacrificed, splenocytes and spinal cord infiltrated mononuclear cells were analyzed BrdU incorporation using the BrdU Flow Kit (BD Pharmingen) according to the manufacturer’s instructions.

### ELISA for cytokines detection

Serum and splenocytes were taken at the peak of disease (day 18 p.i.), levels of IFN-γ, IL-2, IL-17A, IL-10, TGF-β and IL-6 in sera or culture supernatants were evaluated using ELISA kit (all from BD Pharmingen) according to the manufacturer’s instructions.

### Real-time PCR

Total RNA was isolated using Trizol reagent (Invitrogen), reverse transcribed, and polymerase chain reaction amplified using specific primers. One-Step Real-time PCR was performed with SYBR Green PCR Reagents (Qiagen) and a Continuous Fluorescence Detection System (MJ Research, USA), according to the manufacturer’s instructions. Relative quantitation of mRNA expression was calculated as the fold increase in expression by using the ΔΔCt method and the house keeping gene is GAPDH.

### *In vitro* CD4^+^ T cell differentiation

Naïve CD4^+^CD44^-^CD62L^+^ T cells were activated with anti-CD3 (5 µg/ml, BD Pharmingen) and anti-CD28 (2 µg/ml, BD Pharmingen). To induce differentiation into Treg, TGF-β1 (5 ng/ml, Peprotech) and recombinant mouse IL-2 (200 units/ml, BD Pharmingen) were added to the culture. To induce differentiation into Th1, IL-12 (10 ng/ml, Peprotech) and anti-mIL-4 (10 µg/ml, BD Pharmingen) were added to the culture. To induce differentiation into Th17, IL-6 (20 ng/ml, BD Pharmingen), TGF-β1 (5 ng/ml), IL-23 (20 ng/ml, eBioscience), IL-1β (1ng/ml, BD Pharmingen) and anti-mIFN-γ (20 µg/ml, BD Pharmingen) were added to the culture [59]. SM934 (1 µM, final) was added simultaneously.

### Statistical analysis

Data is presented as the mean ± SEM. Comparisons between treated and control groups were made by Student’s unpaired *t-*test after the determination of variances equal by F-test. Statistical difference was accepted at *P* below 0.05.
